# Fatal Myocarditis in Drug Reaction With Eosinophilia and Systemic Symptoms (DRESS) Without Mortality-Related Risk Factors: A Case Report and Literature Review

**DOI:** 10.7759/cureus.63541

**Published:** 2024-06-30

**Authors:** Aya Watanabe, Yohei Nakamoto, Tetsuro Aita, Toru Naganuma, Sei Takahashi, Yuichiro Kiko, Hiroaki Nakagawa, Sugihiro Hamaguchi

**Affiliations:** 1 Department of General Internal Medicine, Fukushima Medical University, Fukushima, JPN; 2 Department of General Internal Medicine, Futaba Emergency and General Medicine Support Center, Fukushima Medical University, Fukushima, JPN; 3 Department of Diagnostic Pathology, Fukushima Medical University, Fukushima, JPN; 4 Department of General Internal Medicine, Center for Innovative Research for Communities and Clinical Excellence, Fukushima Medical University, Fukushima, JPN

**Keywords:** hhv6, drug reaction with eosinophilia and systemic symptoms (dress), mortality-related risk factors, latent period, myocardial biopsy, eosinophilic myocarditis

## Abstract

Drug reaction with eosinophilia and systemic symptoms (DRESS) is a severe drug reaction characterized by skin rash, organ involvement, lymph node swelling, eosinophilia, and atypical lymphocytosis, with myocarditis being a rare but potentially fatal complication. It has been reported that in patients with cardiac involvement due to DRESS, older age and shorter periods between offending drug exposure and symptom onset are associated with mortality. We report a case of fatal DRESS-associated myocarditis in a young woman, occurring one month after drug exposure, despite intensive immunosuppressive therapy. This case report highlights the risk of mortality from DRESS-associated myocarditis even in patients lacking known risk factors.

## Introduction

Drug reaction with eosinophilia and systemic symptoms (DRESS) is a severe hypersensitivity reaction to drugs, typically manifesting within two to six weeks after exposure. It presents with erythema, eosinophilia, atypical lymphocytosis, lymphadenopathy, and organ involvement. The overall population risk was estimated to be between one in 1,000 and one in 10,000 drug exposures [1]. Approximately 90% of patients with DRESS have organ involvement [2], and 4%-21% have cardiac involvement [3]. DRESS-associated myocarditis presents in two forms: acute hypersensitivity myocarditis (AHM) and acute necrotizing eosinophilic myocarditis (ANEM). While AHM typically resolves after discontinuation of the offending drug, ANEM carries a mortality rate of approximately 50% [4]. Interestingly, among patients with cardiac involvement, one-third developed symptoms later during the course, while two-thirds presented at initial diagnosis [5]. Regarding treatment, systemic glucocorticoids are the primary therapy for cardiac involvement. Additional immunosuppressive agents such as cyclosporine, immunoglobulin therapy, mycophenolate mofetil, and mepolizumab have shown efficacy in sparing steroids; however, no standard medication regimen has been established [6,7]. Despite the potential risk of fatal cardiac complications, there is no established prognostic score, and the risk factors for progression to cardiac complications remain unknown. However, a systematic review reported that older age and short latency from drug exposure to symptom onset were associated with mortality due to DRESS-associated myocarditis.

In this report, we present the case of a young, healthy woman with no mortality-related risk factors. The patient developed DRESS-associated myocarditis, which was fatal despite young age and the long duration between drug exposure and symptom onset.

## Case presentation

A 30-year-old woman with irritable bowel syndrome (IBS) was admitted to a local hospital with fever and erythema. Following admission, she developed eosinophilia, atypical lymphocytosis, lymphadenopathy, and facial edema, suggestive of a DRESS. Suspected of DRESS and receiving prednisolone (PSL) at a dosage of 55 mg daily, she was subsequently transferred to our hospital for further management.

Upon admission, she presented with a fever, tender cervical lymphadenopathy, erythema on the anterior chest, skin exfoliation on the trunk and extremities, and petechiae on the palms. Polymerase chain reaction (PCR) testing for human herpes virus 6 (HHV6) was performed. The results of the serum quantitative PCR for HHV-6 were 2,000,000 copies/mL. According to the European Registry of Severe Cutaneous Adverse Reactions (RegiSCAR) criteria [2], the DRESS score was seven. Upon further medical history review, it was found that she had developed relatively severe abdominal pain during several days of intense work and was diagnosed with an exacerbation of IBS at her clinic one month before the rash onset. At that time, she was prescribed mepenzolate bromide combined with phenobarbital, an anticholinergic and antiepileptic agent. The drug-induced lymphocyte stimulation test (DLST) result for phenobarbital was positive, confirming a diagnosis of phenobarbital-induced DRESS. Electrocardiography showed no signs of myocarditis, and troponin I levels were within normal limits.

**Figure 1:**
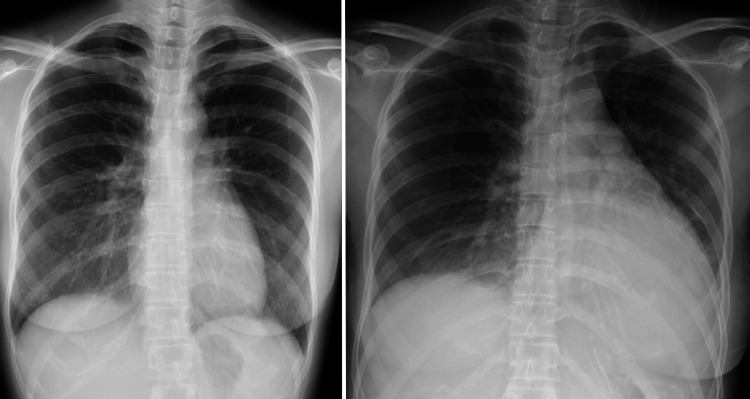
Chest X-ray Chest radiograph at the onset of DRESS (left). Chest radiograph obtained at the onset of initial heart failure, showing cardiomegaly with pulmonary congestion (right). DRESS: Drug reaction with eosinophilia and systemic symptoms.

When tapering oral PSL to 50 mg daily, the patient experienced a relapse characterized by fever, erythema, and eosinophilia. She subsequently underwent pulse therapy with intravenous methylprednisolone (mPSL) at a dosage of 500 mg/day for three consecutive days, followed by oral PSL at 50 mg/day and cyclosporine at 200 mg/day, resulting in resolution of fever, erythema, and eosinophilia. However, two months after the onset of DRESS and tapering of PSL to 20 mg daily, the patient presented with nausea, vomiting, and malaise, prompting evaluation at the local hospital. While free of fever, erythema, or eosinophilia, chest radiography revealed cardiomegaly with pulmonary congestion (Figure 1), prompting suspicion of acute congestive heart failure and subsequent transfer to our hospital.

**Figure 2:**
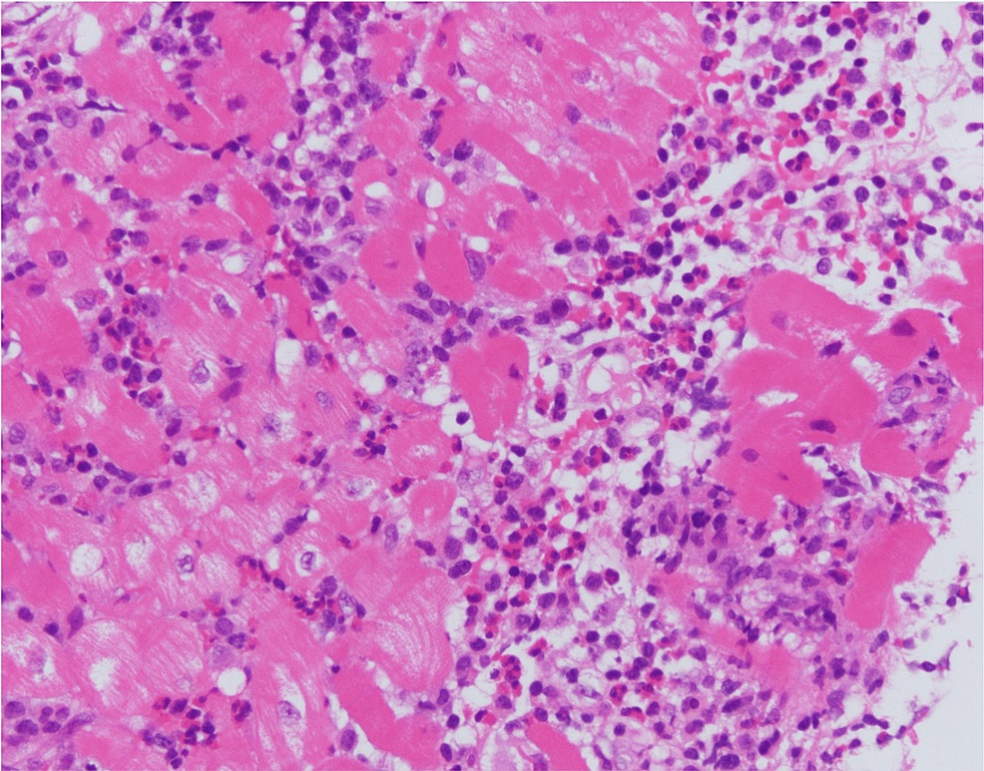
Endomyocardial biopsy Histopathology examination reveals infiltration of eosinophils and macrophage cells between muscle fibers, indicative of eosinophilic myocarditis.

Echocardiography revealed diffuse left ventricular wall hypokinesis, left ventricular hypertrophy, and a small pericardial effusion. Laboratory studies showed elevated levels of troponin I and brain natriuretic peptide (BNP), alongside abnormal liver function. Emergency coronary angiography ruled out coronary artery stenosis. However, rapid examination of the myocardial biopsy revealed infiltration of numerous eosinophils and inflammatory cells between myocardial cells (Figure 2), leading to a diagnosis of eosinophilic myocarditis secondary to DRESS.

Treatment commenced with pulse therapy, administering 1000 mg of intravenous mPSL, followed by a daily oral PSL dose of 60 mg. Diuretics were also administered, resulting in improved pulmonary congestion and reduced troponin I levels. Upon tapering the PSL dose to 40 mg/day, the patient was discharged from our hospital.

The PSL was carefully tapered each month, and her cardiac enzymes were not elevated. However, eight months after the onset of DRESS, troponin I levels gradually increased when PSL was tapered to 25 mg daily, despite the absence of heart failure symptoms. She was readmitted to our hospital, and a myocardial biopsy revealed marked eosinophilic infiltration, confirming recurrent eosinophilic myocarditis. Treatment included mPSL pulse therapy and subcutaneous administration of mepolizumab, a monoclonal antibody targeting human interleukin-5, at a dose of 300 mg/day. Nonetheless, troponin I levels increased again within a week, accompanied by a new complete right bundle branch block observed on electrocardiography. Further intervention involved another round of mPSL pulse therapy and the addition of 750 mg of intravenous cyclophosphamide. Despite these efforts, echocardiography revealed a decreased left ventricular wall motion.

**Figure 3:**
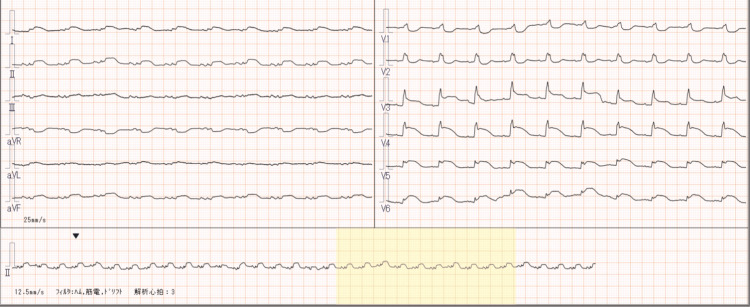
Electrocardiography Electrocardiography at the time of exacerbation of myocarditis showing ST elevation in leads I, II, and V3-6.

**Figure 4:**
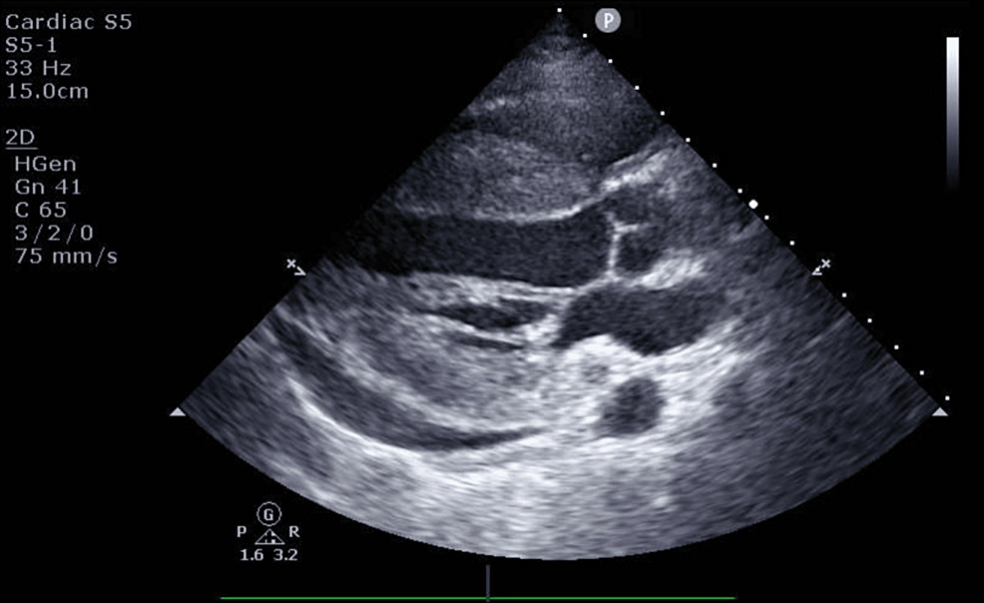
Transthoracic echocardiography Echocardiography at the time of exacerbation of myocarditis showed left ventricular hypertrophy and pericardial effusion.

Fifty-two days after readmission, the patient developed severe chest and back pain, accompanied by ST elevation in leads I, II, and V3-6 on electrocardiography (Figure 3), and left ventricular hypertrophy and pericardial effusion on echocardiography (Figure 4). Troponin I levels were markedly elevated. Emergency coronary angiography revealed a normal coronary artery; she was in cardiogenic shock and had treatment with catecholamines and extracorporeal membrane oxygenation. However, her circulatory dynamics did not stabilize, and she succumbed.

## Discussion

In this report, we present a case of DRESS-associated myocarditis in a previously healthy 30-year-old woman, who followed a fatal course despite intensive immunosuppressive therapy.

DRESS is a severe drug hypersensitivity reaction characterized by a widespread skin rash accompanied by organ involvement, fever, lymphadenopathy, eosinophilia, and atypical lymphocytosis [2]. While the detailed pathogenesis of the disease remains unclear, it is thought to involve drug-specific immune responses, including an expansion of CD4+ T cells, and reactivation of herpes viruses such as HHV6 [8,9]. In our case, the patient exhibited typical symptoms of DRESS, including a skin rash, fever, lymphadenopathy, eosinophilia, and an increased number of atypical lymphocytes. However, the initial diagnosis was challenging due to the unidentified offending drug. We obtained an additional history of drug prescriptions and found that she was taking a drug for IBS containing phenobarbital, one of the most common offending drugs. Therefore, we strongly suspected DRESS as the cause of the manifestations and started further evaluations, such as DLST or PCR, for HHV6. The DLST for phenobarbital was positive, and HHV6 was detected using PCR.

Identifying the offending drug is crucial, as it is found in approximately 80% of DRESS cases (antiepileptic drugs, 35%; allopurinol, 18%; antimicrobial sulfonamides and dapsone, 12%; and other antibiotics, 11%). However, the offending drugs are unclear in 10%-20% of cases, and no drug exposure is present in 2% of cases [2].

Eosinophilic myocarditis represents one of the most severe complications associated with DRESS. Cationic proteins, such as major basic proteins, eosinophil cationic proteins, eosinophil-derived neurotoxins, and eosinophil peroxidases, present in specific eosinophilic granules and can function as cardiotoxic agents, leading to necrosis and apoptosis upon their release from the myocardium [3]. Histological examination of cardiac biopsies often reveals inflammatory cell infiltration, predominantly consisting of eosinophils and lymphocytes, suggesting a direct role of cytotoxic T cells in damaging cardiac tissue [3]. While advanced age (>65 years) and a short duration (<15 days) from drug exposure to symptom onset have been reported as poor prognostic factors for cardiac involvement in DRESS [5], our patient, a 30-year-old woman who developed the disease approximately one month after phenobarbital intake, did not exhibit any of these factors. Nevertheless, despite receiving multiple intensive immunosuppressive therapies, the patient failed to respond and succumbed to fulminant myocarditis.

In a literature review of young patients aged 30 years or younger who died of DRESS-associated myocarditis, we identified only five previously reported cases (Table 1). Among these cases, two had a shorter latency period, whereas three had a longer latency period (>15 days). For instance, one patient developed DRESS four weeks after receiving minocycline, leading to giant cell myocarditis on myocardial biopsy [10]. Despite undergoing heart transplantation, the patient ultimately died of graft dysfunction, unlike the present case, where the cause of death was unrelated to DRESS-associated myocarditis. The other two cases differ from the present case in which they experienced rapid deterioration or sudden death at the onset of myocarditis, precluding the use of immunosuppressive drugs such as steroids [11,12].

**Table 1:** Clinical characteristics of patients dying under 30 years of age from DRESS-associated myocarditis Time 1: Time from drug exposure to the onset of disease. Time 2: Time from the onset of DRESS to the onset of myocarditis. MINO: Minocycline; SSZ: Sulfasalazine; ABPC: Ampicillin; PB: Phenobarbital; DRESS: Drug reaction with eosinophilia and systemic symptoms; mPSL: methylprednisolone; MFM: Mycophenolate mofetil.

Author	Age	Sex	Drug	Time 1	Time 2	Treatment	Primary disease
Jeremic et al. [13]	28 years	Male	SSZ	13 days	At the onset of DRESS	After the onset of myocarditis: mPSL	Hyper IgD syndrome
deMello et al. [11]	14 years	Male	ABPC	7 days	At the onset of DRESS	No immunosuppressive drugs	Unknown
Micozzi et al. [10]	20 years	Female	MINO	4 weeks	4 weeks	Before the onset of myocarditis: steroid; after the onset of myocarditis: mPSL, MFM, anti-thymocyte globulin	Acne
deMello et al. [11]	22 months	Female	ABPC or PB	1.5 months	2 weeks	No immunosuppressive drugs	Meningitis
Parneix-Spake et al. [12]	15 years	Male	MINO	1 month	2.5 months	Before the onset of myocarditis: steroid	Unknown
Our case	30 years	Female	PB	1 month	2 months	Before the onset of myocarditis: steroid, cyclosporine; after the onset of myocarditis: steroids, cyclosporine, cyclophosphamide, mepolizumab	IBS

Our case illustrates the refractory nature of DRESS-associated myocarditis, emphasizing its potential to be life-threatening even in the absence of known risk factors such as older age and a short latency period. Hence, close monitoring of cardiac involvement in DRESS patients is essential. Patients and their families across all age groups must be informed about the risk of fatal cardiac complications, even in the absence of identifiable risk factors. Further research is warranted to assess the risk of complications associated with cardiac involvement and to explore treatment options.

## Conclusions

In our case, a young woman succumbed to DRESS-associated myocarditis due to a poor response to immunosuppressive drugs. Our case illustrates that DRESS-associated myocarditis can be fatal even in patients without known mortality risk (older age and shorter latency period between drug exposure and DRESS symptom onset). Therefore, careful monitoring of cardiac involvement is essential for patients with DRESS. Further research is warranted to determine the risk of complications and optimize treatment strategies for cardiac involvement.
